# Limited Substrate Specificity of PS/γ-Secretase Is Supported by Novel Multiplexed FRET Analysis in Live Cells

**DOI:** 10.3390/bios11060169

**Published:** 2021-05-26

**Authors:** Mei C. Q. Houser, Yuliia Turchyna, Florian Perrin, Lori Chibnik, Oksana Berezovska, Masato Maesako

**Affiliations:** 1 Alzheimer Research Unit, MassGeneral Institute for Neurodegenerative Disease, Massachusetts General Hospital, Harvard Medical School, 114, 16th Street, Charlestown, MA 02129, USA; mhouser@mgh.harvard.edu (M.C.Q.H.); yturchyna@mgh.harvard.edu (Y.T.); fperrin@mgh.harvard.edu (F.P.); lchibnik@hsph.harvard.edu (L.C.); oberezovska@mgh.harvard.edu (O.B.); 2 Department of Epidemiology, Harvard T.H. Chan School of Public Health, 677 Huntington Ave., Boston, MA 02115, USA

**Keywords:** PS/γ-secretase, FRET, multiplexing, APP C99, Notch1 N100

## Abstract

Presenilin (PS)/γ-secretase is an aspartyl protease that processes a wide range of transmembrane proteins such as the amyloid precursor protein (APP) and Notch1, playing essential roles in normal biological events and diseases. However, whether there is a substrate preference for PS/γ-secretase processing in cells is not fully understood. Structural studies of PS/γ-secretase enfolding a fragment of APP or Notch1 showed that the two substrates engage the protease in broadly similar ways, suggesting the limited substrate specificity of PS/γ-secretase. In the present study, we developed a new multiplexed imaging platform that, for the first time, allowed us to quantitatively monitor how PS/γ-secretase processes two different substrates (e.g., APP vs. Notch1) in the same cell. In this assay, we utilized the recently reported, spectrally compatible visible and near-infrared (NIR)-range Förster resonance energy transfer (FRET) biosensors that permit quantitative recording of PS/γ-secretase activity in live cells. Here, we show that, overall, PS/γ-secretase similarly cleaves Notch1 N100, wild-type APP C99, and familial Alzheimer’s disease (FAD)-linked APP C99 mutants in Chinese hamster ovary (CHO) cells, which further supports the limited PS/γ-secretase substrate specificity. On the other hand, a cell-by-cell basis analysis demonstrates a certain degree of variability in substrate recognition and processing by PS/γ-secretase among different cells. Our new multiplexed FRET assay could be a useful tool to better understand how PS/γ-secretase processes its multiple substrates in normal and disease conditions in live, intact cells.

## 1. Introduction

γ-Secretase is an aspartyl protease complex [1,2] in which Presenilin (PS) acts as the catalytic subunit [3]. The PS/γ-secretase complex was first discovered by its association with Alzheimer’s disease (AD); dominantly inherited mutations that resulted in early onset of AD were identified on the genes encoding PS [4,5] and its substrate, the amyloid precursor protein (APP) [6]. Cleavage of the N-terminally clipped APP (i.e., C99) by PS/γ-secretase results in the generation of amyloid β (Aβ) peptides [1]. The accumulation of such Aβ peptides in the brain, so-called neuritic plaques, is one of pathological hallmarks of AD. Since then, numerous other membrane-associated substrates of PS/γ-secretase have been uncovered [7,8], including Notch1, a gene crucial for neurogenesis as well as skeletal and vasculature formation in development [9,10,11]. Moreover, an increasing number of mutations that result in Acne inversa/hidradenitis suppurativa (HS), a chronic, recurrent inflammatory disease of the skin, have been discovered on the genes encoding the subunits of the PS/γ-secretase complex [12,13]. Hence, it is undoubted that PS/γ-secretase is a potential therapeutic target; however, there are significant challenges due to the protease’s multifunctional roles in normal health and disease. 

To lower the production of Aβ as a therapeutic strategy for AD, the inhibition of APP cleavage by PS/γ-secretase has been a topic of significant interest. However, prevention of proper Notch1 cleavage and subsequent disruption of the intracellular signaling resulted in undesired adverse events such as skin cancer [14]. Therefore, a closer look into the recognition and processing of various substrates is vital to develop therapeutic approaches targeting PS/γ-secretase. However, whether PS/γ-secretase similarly processes its multiple substrates or in a substrate-specific manner is still not clearly understood. Of note, PS/γ-secretase substrates present low sequence conservation [7,8,15]. Recent cryo-electric microscopy (cryo-EM) studies analyzing the Presenilin 1 (PS1)/γ-secretase-APP C83 or Notch1 N100 complexes discovered that both the protease and substrate alter their structure upon binding [16,17]. The transmembrane domains of PS1 undergo a conformation change to recognize and bind to APP and Notch1. Both of the substrates also undergo a dynamic structural change in the transmembrane helix to form a β-strand upon binding to the enzyme. The key findings from these studies were that the β-strand domain of both substrates binds to the same general location of PS1, and the conformation of γ-secretase remains unchanged between APP and Notch1. These findings support the challenges of PS/γ-secretase’s substrate selective recognition, processing, and therapeutic targets. 

To elucidate how PS/γ-secretase activity is spatiotemporally regulated in live cells, we recently developed Förster resonance energy transfer (FRET) reporter probes—the APP C99 YPet-mTurquoise-GL (C99 Y-T)—and a Notch1-based analog—N100 Y-T biosensors [18]. Using these biosensors, we uncovered that PS/γ-secretase activity is heterogeneously regulated on a cell-by-cell basis in live primary neurons [18]. Furthermore, to enable multiplexed recording of PS/γ-secretase activity along with other essential molecular and cellular events in the same cell, we have substituted YPet and mTurquoise-GL with miRFP720 and miRFP670, respectively, allowing the development of a near-infrared (NIR) C99 miRFP720-miRFP670 (C99 720-670) biosensor [19]. Shcherbakova et al. [20] and then we [19] have validated the spectral compatibility of the NIR fluorescent proteins with cyan-to-yellow color range proteins. In the present study, to gain deeper knowledge regarding how PS/γ-secretase cleaves multiple substrates in intact cells, we utilized the recently developed APP C99 Y-T/Notch1 N100 Y-T [18] and the C99 720-670 probes [19], which permit, for the first time, the co-monitoring of two independent substrates’ processing by PS/γ-secretase on a cell-by-cell basis. Using this new multiplexed assay, we examined how APP C99 and Notch1 N100 are processed by PS/γ-secretase in the same cell. Moreover, we compared the processing efficiency between WT APP C99 and two FAD-linked mutants APP C99, E693G Arctic [21], and the V717I London mutation [22], and also between Notch 1 N100 and the FAD APP C99. We show that Notch1 N100, APP C99, and FAD mutants APP C99 are overall similarly processed on a cell population basis. Nevertheless, some cells process the two different substrates more while others less, demonstrating striking heterogeneity in PS/γ-secretase activity on a cell-by-cell basis. Furthermore, we found a certain degree of variation in the substrate recognition and processing of PS/γ-secretase among the cells, evidenced by the fact that one may be more processed in some cells than the other (i.e., the cell-by-cell heterogeneity in substrate recognition and processing of PS/γ-secretase). Our findings demonstrate the complex nature of PS/γ-secretase on its multiple substrate processing, which could play a role in normal health and disease.

## 2. Materials and Methods

### 2.1. Plasmid Constructs

The N100 YPet-mTurquoise-GL (N100 Y-T), C99 Y-T and C99 miRFP720-miRFP670 (C99 720-670) biosensors were developed and validated in the previous studies [18,19]. Based on the calculation using FPbase (https://www.fpbase.org/fret/) (accessed on 14 May 2021), which is an open-source database for fluorescent proteins and their properties, the Förster radius of mTurquoise-YPet (mTurquoise-GL and YPet has not been elucidated) and miRFP670-miRFP720 is 57.28 Å and 56.94 Å, respectively. To introduce the E693G Arctic mutation to the C99 Y-T and C99 720-670 biosensors, we performed site-directed mutagenesis using the QuikChange site-directed mutagenesis kit (Agilent Technologies, Santa Clara, CA) and the following primers: forward (FW): AAATTGGTGTTCTTTGCAGGAGATGTGGGTTCAAACAAA, reverse (RV): TTTGTTTGAACCCACATCTCCTGCAAAGAACACCAATTT. The V717I London mutant C99 Y-T and C99 720-670 biosensors were developed using the following primers: FW: GTCATAGCGACAGTGATCATCATCACCTTGGTGATGCTG, RV: CAGCATCACCAAGGTGATGATGATCACTGTCGCTATGAC. The sequence of all plasmids was verified by MGH CCIB DNA core.

### 2.2. Cell Culture and Plasmid Transfection

The maintenance of Chinese hamster ovary (CHO) cells and plasmid DNA transfection were performed as described previously [19]. Briefly, Opti-MEM Reduced Serum Medium (Thermo Fisher Scientific, Waltham, MA, USA) with 5% fetal bovine serum (FBS) (Atlanta Biologicals Inc., Flowery Branch, GA, USA) was used for the cell culturing, and Lipofectamine 3000 (Thermo Fisher Scientific, Waltham, MA, USA) was used for plasmid transfection.

### 2.3. Confocal Microscopy and FRET Analysis

Multiplexed FRET analysis was performed as described previously [19]. Briefly, the diode laser (wavelengths of 405 nm and 640 nm) was used to excite mTurquoise-GL in the N100 Y-T, the wild type, or FAD mutant C99 Y-T biosensors and miRFP670 in the wild-type or FAD mutant C99 720-670 biosensors, respectively. The emitted fluorescence was simultaneously detected at 460–480 nm (mTurquoise-GL), 520–540 nm (YPet), 660–680 nm (miRFP670), and 700–720 nm (miRFP720) using the standard (Multi-alkali photomultiplier tube (PMT)) and high-sensitivity (Cooled GaAsP PMT) detectors on an Olympus FV3000RS Confocal Laser Scanning Microscope. A CO2/heating unit (Tokai-Hit STX-Co2 Digital CO2 Gas Mixing System) was used during the imaging. Images were taken with a 10×/0.25 NA objective. Image J was used to measure average pixel fluorescence intensity after the background subtraction (region of interest (ROI): whole cell). As readouts of the FRET efficiency, the emission intensity of YPet over mTurquoise-GL (Y/T) and the miRFP720 emission over miRFP670 (720/670) ratios were used. MATLAB (The MathWorks, Inc., Natick, MA, USA) was used to generate pseudo-colored images. 

### 2.4. Statistical Analysis

GraphPad Prism 9 (GraphPad Software, San Diego, CA, USA) was used to perform statistical analysis in the present study. D’Agostino–Pearson omnibus normality test was used to determine the Gaussian distribution of the data. A one-way analysis of variance (ANOVA) followed by Tukey’s multiple comparison was applied to compare the data. The Pearson correlation coefficient squared and simple linear regression was applied to measure linear correlation and slope. A *p*-value of <0.05 was considered a predetermined threshold for statistical significance.

The calculation of sample size was based on previous results in our laboratory [18,19]. Briefly, 25–30 cells per condition are needed to reach statistical difference in the FRET analysis. All experiments were repeated in four to six independent experiments, the data were combined from all of the experiments, and the number of biological replicates was shown. All values are given as means ± standard deviation (SD).

## 3. Results

### 3.1. Co-Recording of APP C99 and Notch 1 N100 Processing by PS/γ-Secretase on a Cell-by-Cell Basis in Live Cells

While a wide variety of transmembrane proteins have been identified as substrates of PS/γ-secretase, little is known about how this protease cleaves its multiple substrates in intact cells. To quantitatively record the processing of two independent substrates by PS/γ-secretase on a cell-by-cell basis in live cells, we utilized our previously developed YPet/mTurquoise-GL and miRFP720/miRFP670-based FRET biosensors. The changes in the acceptor over donor emission ratio (i.e., FRET efficiency) can be used as a readout of changes in PS/γ-secretase activity; a decrease in overall ratios evidences the activation of PS/γ-secretase [18,19]. The visible and NIR range fluorescent proteins in these biosensors are spectrally compatible [19], and either APP C99 or Notch1 N100 can be selected as the immediate substrate domain of the biosensors [18]. Therefore, multiplexed FRET recording of the two different biosensors enables quantitative monitoring of the PS/γ-secretase processing of two different substrates in the same cell. 

**Figure 1:**
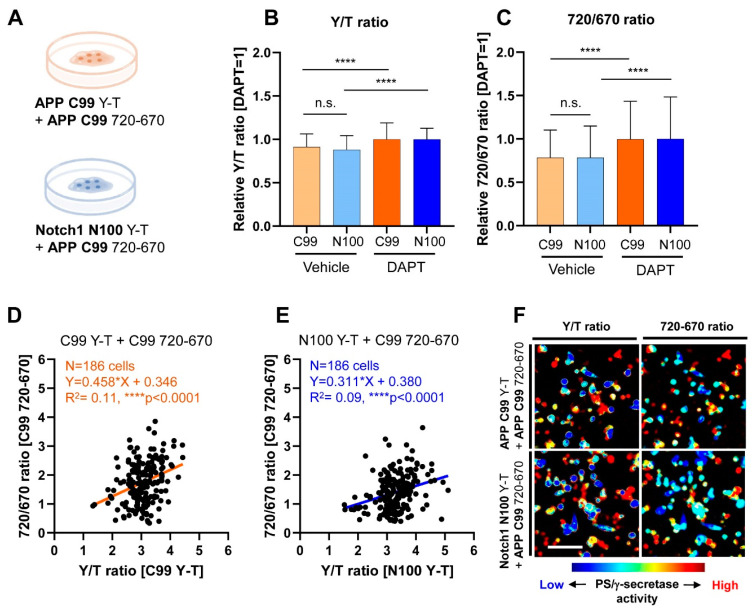
Co-recording of APP C99 and Notch1 N100 processing by PS/γ-secretase on a cell-by-cell basis in live cells: (**A**) A schematic presentation of the experimental conditions. The APP C99 720-670 biosensor was co-transfected with either the APP C99 Y-T or Notch1 N100 Y-T biosensor in CHO cells; (**B**) CHO cells co-expressing the C99 720-670 with C99 Y-T biosensors or those with the N100 Y-T biosensors were treated with 1 μM DAPT (PS/γ-secretase inhibitor) or vehicle control for 16 h. Y/T ratios in the cells expressing the C99 Y-T biosensor were normalized to those in the corresponding DAPT-treated cells and similarly done for the cells expressing the N100 Y-T biosensors to calculate relative Y/T ratios, which indicate the processing efficiency of the reporter probe by PS/γ-secretase. The relative Y/T ratio was not different between the cells co-expressing the C99 720-670 with C99 Y-T biosensor and those with the N100 Y-T in vehicle-treated condition. *n* = 186–188, one-way ANOVA, n.s. not significant, **** *p* < 0.0001; (**C**) In accordance, relative 720/670 ratios remained largely unchanged in the presence of either C99 Y-T or N100 Y-T biosensors, suggesting that the C99 720-670 biosensor was equally processed by PS/γ-secretase in these cells. *n* = 186–188, one-way ANOVA, n.s. not significant, **** *p* < 0.0001; (**D**) Scatterplots of 720/670 and Y/T ratios in the cells expressing the C99 720-670 and C99 Y-T biosensors (Pearson correlation coefficient, *n* = 186, Y = 0.458X + 0.346, R^2^ = 0.11, **** *p* < 0.0001) or (**E**) those with the C99 720-670 and the N100 Y-T biosensors (*n* = 186, Y = 0.311X + 0.380, R^2^ = 0.09, **** *p* < 0.0001). (**F**) Pseudo-colored images of Y/T ratio and 720/670 ratio: cells with higher and lower PS/γ-secretase activity are shown in red and blue, respectively.

First, to examine whether PS/γ-secretase similarly or differently processes APP C99 and Notch1 N100, CHO cells were co-transfected with either the APP C99 Y-T or the Notch1 N100 Y-T biosensor combined with the C99 720-670 biosensor (Figure 1A). CHO cells have been widely used as one of the cell models since the line is well validated to express endogenous functional PS/γ-secretase [23,24]. The cells were then treated with vehicle or 1 μM DAPT, a known PS/γ-secretase inhibitor [25], for 16 h. Live imaging of the cells via confocal microscopy was performed approximately 30 h post-transfection. In the spectral FRET analysis, the cells were excited using 405 nm and 640 nm lasers. The emitted fluorescence was simultaneously detected by four independent channels that allow measurement of the emission of mTurquoise-GL, YPet, miRFP670, and miRFP720. The YPet over mTurquoise-GL (Y/T) ratios in the cells treated with vehicle control were calculated and normalized to the corresponding ratios of DAPT-treated cells; this was repeated for the 720/670 ratios. The significantly lower ratios of Y/T and 720/670 in vehicle-treated cells compared to those in DAPT demonstrate that the biosensors were processed by PS/γ-secretase, and changes in PS/γ-secretase activity were accurately reported (Figure 1B,C) [18,19]. Importantly, we found that the average Y/T ratio in cells expressing the C99 Y-T biosensor was similar to that of cells expressing the N100 Y-T in the vehicle treatment condition (Figure 1B). In accordance, 720/670 ratios also showed no significant difference in the cells where the C99 720-670 biosensor was co-transfected with the C99 Y-T biosensor compared to those with the N100 Y-T biosensor (Figure 1C). These results clearly suggest that, overall, PS/γ-secretase similarly processes APP C99 and Notch1 N100 on a cell population basis in living CHO cells. 

To further understand how PS/γ-secretase processes the two substrates, we analyzed the scatterplots charting the Y/T and 720/670 ratios of individual cells. We found a statistically significant, positive correlation between the Y/T and 720/670 ratios among in cells co-expressing the C99 Y-T (Figure 1D) or the N100 Y-T (Figure 1E) with the C99 720-670 biosensors. Cells with lower Y/T ratios overall displayed lower 720/670 ratios, indicating more processing of both substrates by PS/γ-secretase in the same cell, while higher Y/T ratios also show higher 720/670 ratios. Such a relationship was further evidenced by pseudo-color images of the Y/T and 720/670 ratios on a cell-by-cell basis (Figure 1F), indicating that PS/γ-secretase activity is heterogeneously regulated among cells [18]. Although the Pearson correlation coefficient squared shows a significant positive correlation, the low R^2^ scores in the analysis suggest that one substrate is processed more than another in a particular cell (Figure 1D,E), demonstrating a certain degree of variability in the recognition and processing of substrates by PS/γ-secretase on a cell-by-cell basis.

### 3.2. Simultaneous Monitoring of WT and FAD-Linked Mutant APP C99 Processing by PS/γ-Secretase

**Figure 2:**
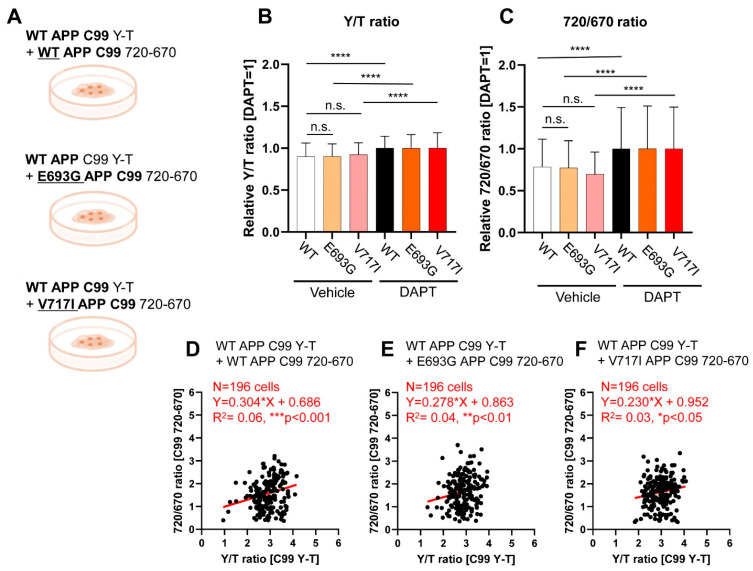
Simultaneous monitoring of WT and FAD-linked mutants APP C99 processing by PS/γ-secretase: (**A**) Schematic representation of the experimental groups: the WT APP C99 Y-T biosensor was co-transfected into CHO cells in combination with the WT or FAD mutant C99 720-670 biosensors; (**B**) The relative Y/T ratio was comparable among three groups, suggesting that the C99 Y-T biosensor was equally processed in the presence of WT or FAD mutants C99 720-670 biosensors. *n* = 196, one-way ANOVA, n.s. not significant, **** *p* < 0.0001; (**C**) Similarly, relative 720/670 ratio was not different between WT versus mutants C99 720-670 biosensors expressing cells. *n* = 196, one-way ANOVA, n.s. not significant, **** *p* < 0.0001. Scatterplots of 720/670 and Y/T ratios in the cells expressing the C99 Y-T biosensor together with the WT C99 720-670 (*n* = 196, Y = 0.304X + 0.686, R^2^ = 0.06, *** *p* < 0.001) (**D**); the E693G C99 720-670 (*n* = 196, Y = 0.278X + 0.863, R^2^ = 0.04, ** *p* < 0.01) (**E**); or the V717I C99 720-670 biosensor (*n* = 196, Y = 0.230X + 0.952, R^2^ = 0.03, * *p* < 0.05) (**F**). Pearson correlation coefficient squared demonstrates a positive correlation between the Y/T ratio (representing the C99 Y-T processing) and 720/670 ratio (cleavage of the wild-type or FAD mutants C99 720-670).

Next, we asked if FAD APP mutations influence the ε-site processing of C99 by PS/γ-secretase. To examine if the mutation location, either associated closely with the PS/γ-secretase cleavage site or not, would contribute to a change in the processing of C99, we chose to use E693G (Arctic) [21] and V717I (London) [22] FAD-linked APP mutants, respectively. The E693G mutation is found on the APP portion located in the extracellular region, while the V717I is located at the PS/γ-secretase cleavage site. To compare the processing efficiency between wild-type and the mutants APP C99, CHO cells were co-transfected with the wild-type C99 Y-T biosensor and either the wild-type form or a mutated version of the C99 720-670 biosensor, containing E693G or V717I mutations (Figure 2A). The cells were then treated with either vehicle or 1 μM DAPT for 16 hours before imaging, and relative Y/T and 720/670 ratios were calculated as described above. 

We found comparable relative Y/T ratios among the cells co-expressing the C99 Y-T biosensor with wild-type, E693G, or V717I C99 720-670 biosensor (Figure 2B), suggesting that wild-type C99 processing is not significantly affected by the presence of FAD mutant APP. The 720/670 ratio also showed no significant difference among the three APP C99 substrates (Figure 2C), which demonstrates that wild-type and the mutant APP C99 are overall similarly processed by PS/γ-secretase. Pearson correlation coefficient squared between the Y/T and 720/670 ratios of all three groups showed a statistically significant, positive correlation (*p*-value < 0.05) (Figure 2D–F). This demonstrates that both wild-type and mutant APP C99 720-670 biosensors are actively processed in some cells but less in others. The low R^2^ values in the correlation analysis reflect variability in the substrate recognition and processing by PS/γ-secretase among the cells, with either wild-type or mutant APP being processed more than the other type (Figure 2D–F). To ensure that these results were not dependent on the biosensor’s fluorophores, mutant versions of C99 Y-T, containing E693G or V717I mutations, were transfected into CHO cells with wild-type C99 720-670 (Figure S1). Both combinations of the biosensors and FAD-linked C99 mutations yielded the same results; wild-type and mutants were similarly processed, further supporting our findings that wild-type and FAD C99 are overall similarly processed and there is a certain degree of heterogeneity in cellular PS/γ-secretase activity, substrate recognition, and processing among the cells.

### 3.3. Co-Monitoring of Notch1 N100 and FAD-Linked Mutant APP C99 Processing by PS/γ-Secretase

**Figure 3:**
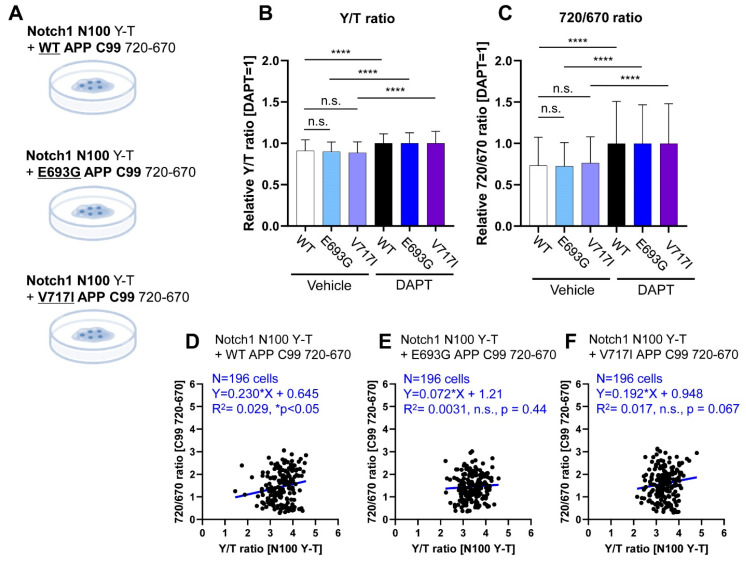
Co-monitoring of Notch1 N100 and FAD-linked mutants APP C99 processing by PS/γ-secretase. (**A**) Schematic representation of the experimental conditions: the Notch1 N100 Y-T biosensor was co-transfected into CHO cells with the WT or FAD mutants C99 720-670 biosensors; (**B**) There is no significant difference in the Y/T ratios of N100 Y-T biosensor in the presence of FAD mutants E693G or V717I C99 720-670 probes. *n* = 196, one-way ANOVA, n.s. not significant, **** *p* < 0.0001.; (**C**) Similarly, there is no change between the processing of WT versus the FAD mutants C99 720-670. *n* = 196, one-way ANOVA, n.s. not significant, **** *p* < 0.0001. Scatterplot of Y/T ratios with 720/670 ratios in the cells expressing the N100 Y-T and (**D**) WT C99 720-670 (*n* = 196, Y = 0.23X + 0.645, R^2^ = 0.029, * *p* < 0.05); (**E**) E693G C99 720-670 (*n* = 196, Y = 0.072X + 1.21, R^2^ = 0.003, n.s. *p* = 0.44); or (**F**) V717I C99 720-670 biosensors (*n* = 196, Y = 0.192X + 0.948, R^2^ = 0.017, n.s. *p* = 0.067).

Lastly, we examined the relationship between Notch 1 N100 and FAD-linked mutant APP C99 (Figure 3A) to further validate that 1) PS/γ-secretase similarly processes two independent substrates and 2) there is variability in PS/γ-secretase substrate recognition and processing among different cells. We found that both relative Y/T and 720/670 ratios in cells co-transfected with the N100 Y-T and the wild-type or mutant C99 720-670 were not significantly different (Figure 3B,C). These results indicate that, overall, PS/γ-secretase similarly processes Notch1 N100 and FAD mutants APP C99. Although the scatterplots of 720/670 versus Y/T ratios show a positive correlation, the low R^2^ indicates some heterogeneity in overall cellular PS/γ-secretase activity as well as substrate recognition or processing by PS/γ-secretase among different cells (Figure 3D–F).

## 4. Discussion

During the past two decades, tremendous effort has allowed the elucidation of the pivotal role of PS/γ-secretase in essential biological events and diseases. To better understand the spatiotemporal regulation of PS/γ-secretase activity in live/intact cells, we engineered genetically encoded FRET-based biosensors that permit the recording of PS/γ-secretase activity over time on a cell-by-cell basis without disrupting the normal environment of cells [18,19]. Here, we report the development of a new multiplexed FRET imaging assay that enables, for the first time, simultaneous measurement of two independent substrates’ cleavage by PS/γ-secretase in the same cell. 

Given that the cleavage of a specific substrate by PS/γ-secretase may play a role in each disease pathogenesis (e.g., APP in AD and Notch receptors in cancers, etc.), substrate-selective inhibition and/or activation of PS/γ-secretase would be desired for the development of efficient therapeutic strategies. However, whether there is a preferential PS/γ-secretase processing of the different substrates, such as APP and Notch1 in particular, in intact cells is still poorly understood. Several pharmacological compounds that potentially block PS/γ-secretase cleavage of APP while sparing Notch processing have been reported [26,27]. However, PS/γ-secretase substrates do not share a unique sequence motif that would be preferentially cleaved [7,8,15]. Furthermore, recent cryo-EM studies of PS/γ-secretase together with APP or Notch1 suggested that the design of substrate-specific inhibitors is challenging (may not be impossible, though) since the common recognition mechanism by PS/γ-secretase as well as the binding location in the enzyme are shared between APP and Notch1 [16,17]. The data from our FRET-based study also suggest that, overall, PS/γ-secretase similarly processes its immediate substrates APP C99 and Notch1 N100 in live cells, further supporting the limited substrate specificity of PS/γ-secretase. 

Previously developed and widely used biochemical analyses, such as *in vitro* PS/γ-secretase activity assay, have enabled the elucidation of how PS/γ-secretase activity is regulated in a bulk population of cells. Our recent development of FRET-based biosensors reporting PS/γ-secretase activity on a cell-by-cell basis has permitted us to uncover that PS/γ-secretase activity is heterogeneously regulated in different cells [18]. Notably, findings from the present study suggest that two different substrates are more processed in some cells but less in others, further strongly supporting the cell-by-cell heterogeneity in PS/γ-secretase activity. The molecular mechanism(s) behind this heterogeneity in cellular PS/γ-secretase activity is still unclear and needs to be addressed in future studies. Although the proteolytic activity of PS/γ-secretase toward different substrates is comparable in cell populations, the somewhat low R-squared values in our correlation analysis support cell-by-cell variability. This variability could be because PS/γ-secretase can process a limited number of substrates, and thus substrates compete with each other in a single cell. Of note, previous studies reported that different PS/γ-secretase substrates compete with each other for PS/γ-secretase activity [28,29].

Since the first discovery [12], over 50 mutations that include frameshift, nonsense, missense, and splice-site mutations have been identified on the genes encoding PS/γ-secretase components (i.e., PS1, Pen2, and Nicastrin), resulting in familial Acne inversa/hidradenitis suppurativa (HS)—a chronic and recurrent inflammatory skin disease [13]. These mutations result in haploinsufficiency and thus loss of function of PS/γ-secretase, and the impaired PS/γ-secretase–Notch pathway is implicated as the molecular pathogenesis of HS. On the other hand, over 350 missense mutations have been identified on the genes encoding PS1 and PS2 (https://www.alzforum.org/mutations) (accessed on 25 May 2021), resulting in the early onset of FAD. Surprisingly, some of the knock-in mice carrying FAD-linked PS1 mutations such as R278I, C410Y, and/or L435F in a homozygous manner [30,31] copy abnormal developmental phenotypes of PS1 knock-out mice [9,10], suggesting that these mutations could result in a drastic loss of function of PS/γ-secretase. A mystery that remains unresolved is why any HS-related clinical symptoms have never been identified in the FAD PS1 mutation careers. Although these unique AD-linked PS1 mutations and the HS-linked haploinsufficiency causing mutations appear to share a similar consequence in PS/γ-secretase activity, one can hypothesize that some substrates—Notch receptors, in particular—could be differently processed between wild-type and the FAD PS1/γ-secretase in skin and/or inflammatory cells. On the other hand, others could expect a feedback mechanism in these cells expressing the FAD mutant PS1 that increases cellular PS/γ-secretase activity. These hypotheses could be directly tested using the multiplexed FRET assay that we report. 

The recent extensive effort to develop multiplexed imaging platforms has enabled a better understanding of complex biological processes. For instance, complex signaling networks in intact cells, such as a change in kinase activity and second messenger transient responses to stimulations, were elegantly dissected by multiplexed imaging [32,33]. Moreover, emerging NIR fluorescent proteins, combined with newly developed NIR–FRET technologies, have opened a new window to understanding how complex biological processes, including calcium dynamics, signaling pathways, and/or proteolysis, are spatiotemporally regulated in cells [19,20,34,35,36,37]. Given that PS/γ-secretase plays a pivotal role in normal development [9,10] and diseases [4,5,12], multiplexed recording of PS/γ-secretase utilizing the NIR-C99 720-670 probe [19] along with other biological events will provide significant insights into PS/γ-secretase biology and its related diseases. 

In conclusion, we report the development of a new multiplexed imaging assay that enables, for the first time, the quantitative assessment of how PS/γ-secretase processes two different substrates on a cell-by-cell basis in live cells. Using this assay, we uncovered that, overall, PS/γ-secretase similarly processes wild-type APP C99, FAD-linked mutant APP, and Notch1 N100 substrates in intact CHO cells. However, the cell-by-cell imaging capability of this assay also strongly supports heterogeneity in PS/γ-secretase activity among cells. The new multiplexed FRET approach allowed us to uncover a certain degree of variability in different substrate recognition and processing by PS/γ-secretase among cells. Our findings would provide a significant insight into the design of therapeutic strategies targeting PS/γ-secretase.

## Data Availability

Not applicable.

## Supplementary Materials

The following are available online at https://www.mdpi.com/article/10.3390/bios11060169/s1, Figure S1: PS/γ-secretase processing of FAD-linked mutant C99 Y-T and WT C99 720-670.
