# Supplementary material for: Limited Substrate Specificity of PS/γ-Secretase Is Supported by Novel Multiplexed FRET Analysis in Live Cells
Source: Biosensors (Basel). 2021 May 26;11(6):169. doi: 10.3390/bios11060169 (PMC8228125; doi:10.3390/bios11060169)
Supplement: Supplementary file 1 [file biosensors-11-00169-s001.zip › biosensors-1206187-supplementary.pdf]

Supporting information

Limited substrate specificity of PS/γ-secretase is supported by novel multiplexed FRET analysis in live cells

Mei CQ Houser, Yuliia Turchyna, Florian Perrin, Oksana Berezovska and Masato Maesako*

Alzheimer Research Unit, MassGeneral Institute for Neurodegenerative Disease, Massachusetts General Hospital, Harvard Medical School, 114, 16th street, Charlestown, MA 02129

Corresponding Author: Masato Maesako, PhD (mmaesako@mgh.harvard.edu)

Supplemental Figure 1

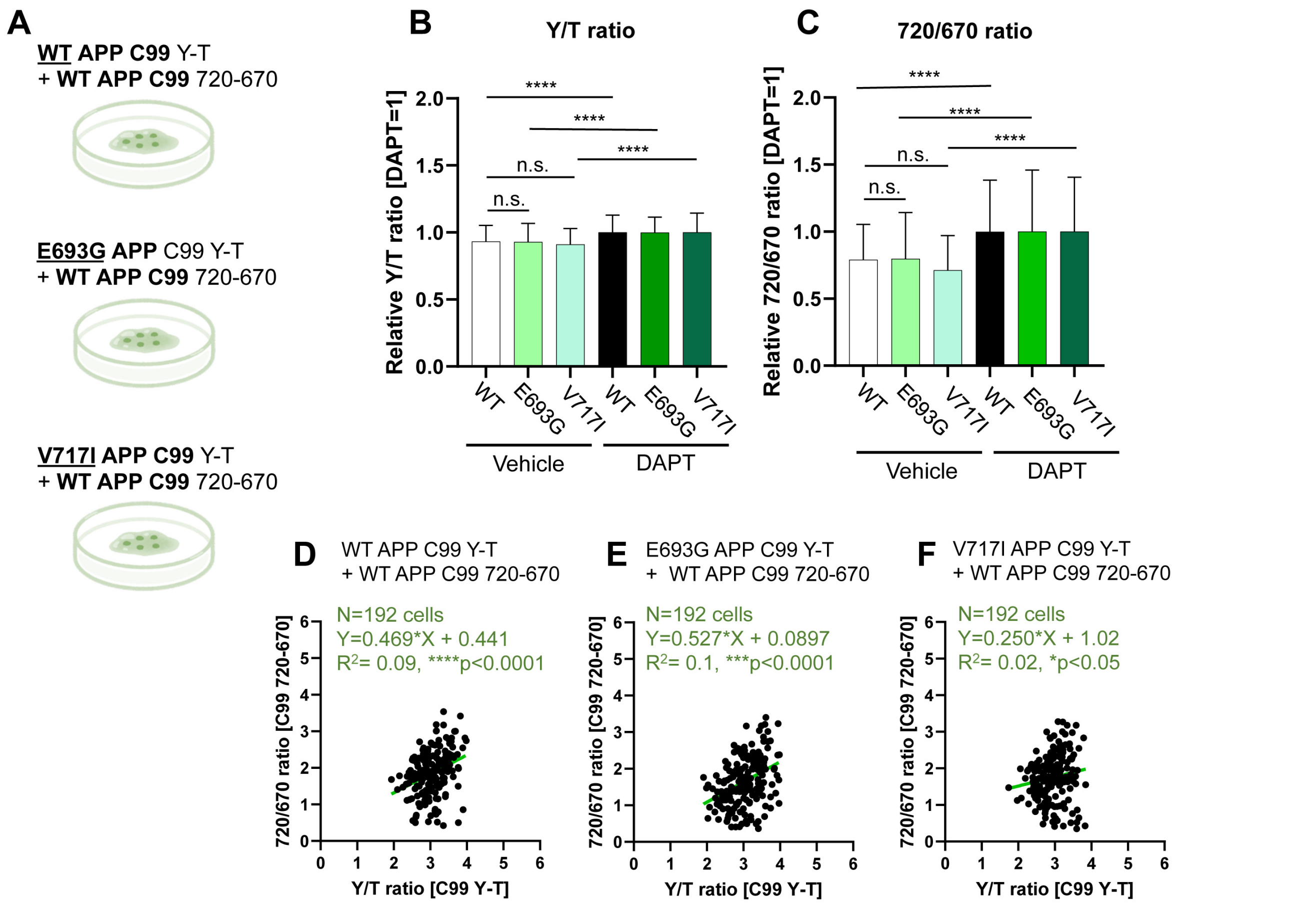

PS/γ-secretase processing of FAD-linked mutant C99 Y-T and WT C99 720-670. (**A**) Schematic representation of the different co-transfections including the WT or FAD C99 Y-T in combination with the WT C99 720-670 biosensors. (**B**) The relative Y/T ratio was similar between all groups, indicating that the WT or FAD C99 Y-T biosensor was equally processed. n=192, one-way ANOVA, n.s. not significant, **** $p < 0.0001$ (**C**) Similarly, relative 720/670 ratio was not different in the presence of WT or mutants C99 Y-T biosensors expressing cells. n=192, one-way ANOVA, n.s. not significant, **** $p < 0.0001$. Scatter plots of 720/670 and Y/T ratios in the cells expressing the C99 Y-T biosensor together with the WT C99 720-670 (n=192, Y = 0.304X + 0.686, R^2 = 0.06, ***p<0.001) (**D**), the E693G C99 720-670 (n=192, Y = 0.277X + 0.863, R^2 = 0.04, **p<0.01) (**E**) or the V717I C99 720-670 biosensor (n=192, Y = 0.229X + 0.952, R^2 = 0.03, *p<0.05) (**F**). Pearson correlation coefficient demonstrates a positive correlation between the Y/T ratio (representing either wild type or FAD mutant C99 Y-T processing) and 720/670 ratio (cleavage of the wild type C99 720-670).
